# Convolutional neural network for automated segmentation of the liver and its vessels on non-contrast T1 vibe Dixon acquisitions

**DOI:** 10.1038/s41598-022-26328-2

**Published:** 2022-12-21

**Authors:** Lukas Zbinden, Damiano Catucci, Yannick Suter, Annalisa Berzigotti, Lukas Ebner, Andreas Christe, Verena Carola Obmann, Raphael Sznitman, Adrian Thomas Huber

**Affiliations:** 1 ARTORG Center for Biomedical Engineering Research, University of Bern, Murtenstrasse 50, 3008 Bern, Switzerland; 2 Department of Diagnostic, Interventional and Pediatric Radiology, Inselspital, Bern University Hospital, Freiburgstrasse 10, 3010 Bern, Switzerland; 3 Hepatology, Department of Visceral Surgery and Medicine, Inselspital, Bern University Hospital, Bern, Switzerland

**Keywords:** Liver, Liver, Liver fibrosis, Biomedical engineering, Computer science

## Abstract

We evaluated the effectiveness of automated segmentation of the liver and its vessels with a convolutional neural network on non-contrast T1 vibe Dixon acquisitions. A dataset of non-contrast T1 vibe Dixon liver magnetic resonance images was labelled slice-by-slice for the outer liver border, portal, and hepatic veins by an expert. A 3D U-Net convolutional neural network was trained with different combinations of Dixon in-phase, opposed-phase, water, and fat reconstructions. The neural network trained with the single-modal in-phase reconstructions achieved a high performance for liver parenchyma (Dice 0.936 ± 0.02), portal veins (0.634 ± 0.09), and hepatic veins (0.532 ± 0.12) segmentation. No benefit of using multi-modal input was observed (p = 1.0 for all experiments), combining in-phase, opposed-phase, fat, and water reconstruction. Accuracy for differentiation between portal and hepatic veins was 99% for portal veins and 97% for hepatic veins in the central region and slightly lower in the peripheral region (91% for portal veins, 80% for hepatic veins). In conclusion, deep learning-based automated segmentation of the liver and its vessels on non-contrast T1 vibe Dixon was highly effective. The single-modal in-phase input achieved the best performance in segmentation and differentiation between portal and hepatic veins.

## Introduction

Medical imaging data is rapidly growing and already constitutes an estimated 90 percent of all healthcare data today^1^. Without technical support, such an overwhelming amount of data cannot be handled by medical experts to provide fast and accurate clinical information about the patient’s health status. In recent years, deep learning (DL) technologies have become the standard for computer vision and imaging tasks^2^. DL technologies with magnetic resonance imaging (MRI) have been used in neuroimaging^3^, but may be used as well in other organs such as the liver, with its complex macro- and microstructure. Besides better image reconstruction algorithms and the detection of focal liver lesions, segmentation tasks of 3D volumetric MRI data represent one of the most promising applications for DL algorithms, notably in diffuse liver disease.

Liver segmentation is fundamental in numerous downstream applications. Focal liver lesions characterization requires exact lesion localization for baseline and follow-up exams. The combination of multiparametric MRI sequences with liver parenchymal and vascular morphology and volumes allows to generate numerous novel quantitative non-invasive MR-biomarkers^4, 5^. Such quantitative biomarkers may improve phenotyping of macro- and microstructural liver remodeling in chronic liver disease, as well as the planning and follow-up of endovascular and surgical liver interventions, including the calculation of the postoperative remnant liver volume (RLV)^6, 7^. However, manual delineation of liver parenchyma, portal, and hepatic veins is time-consuming. An automated method would be needed to perform this task routinely in a reliable and efficient way.

As computed tomography (CT) provides millimetric 3D datasets, it is not surprising that many studies that use DL in abdominal imaging have predominantly been performed using publicly accessible CT images^1, 8^. Due to superior contrast-to-noise ratio, DL algorithms were mostly trained on contrast-enhanced scans^9, 10^. However, many liver imaging studies are nowadays performed by MRI, with advantages in characterizing focal and diffuse liver diseases. Due to its inherent higher contrast-to-noise ratio, an examination without intravenous contrast administration is possible^11^.

Recent studies showed good results with a U-Net-based learning framework on T1 weighted images for automated segmentation of the liver and other abdominal organs ^12^. However, these segmentations only considered the outer liver borders and not the liver veins^13, 14^. For liver vessel segmentation, several results were published using non-DL-based multi-step segmentation approaches on CT images^15^, contrast-enhanced T1 weighted images^16^, non-contrast Fast Imaging with Steady-state Precession (FISP)^17^, and T1 weighted images^18^. The delineation of different veins in the liver and separation of hepatic and portal veins remains a difficult task.

Since pre-contrast T1 weighted acquisitions of the liver are often performed with the Dixon technique, the generated in-phase, and opposed-phase images, including fat- and water-reconstructions, may represent a promising approach as an input for a DL-algorithm. To the best of our knowledge no DL-algorithm for liver parenchyma and vessel segmentation has yet been published on non-contrast T1 weighted Dixon sequences.

The purpose of this study was to evaluate the effectiveness of automated liver parenchyma, portal veins, and hepatic veins segmentation on non-contrast T1 vibe Dixon acquisitions with a convolutional neural network.

## Methods

**Figure 1:**
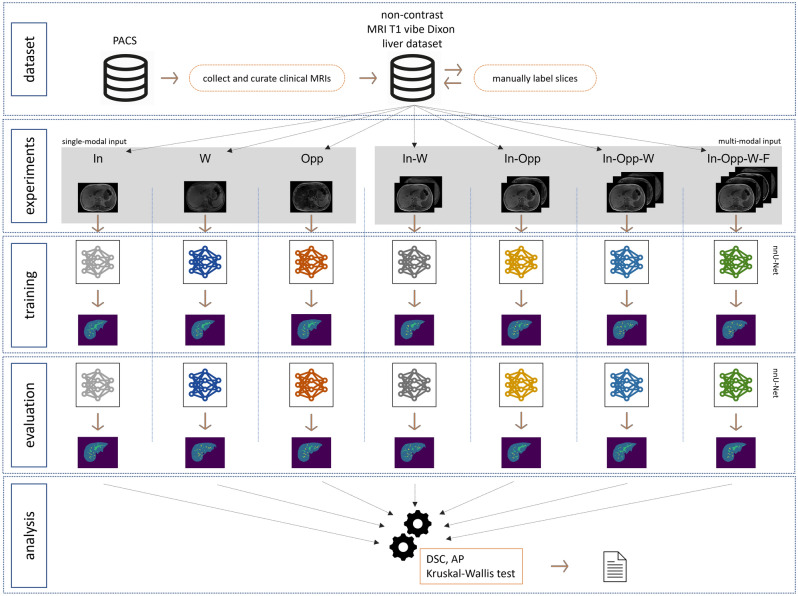
Workflow of our study of automated MRI liver and vessels segmentation with a convolutional neural network (nnU-Net) on non-contrast T1 vibe Dixon acquisitions. The MRI sequences were extracted from a pre-existing picture archiving and communication system (PACS) and manually labelled slice-by-slice. Segmentation experiments with single-modal and multi-modal inputs were defined. In-phase (In), water (W), and opposed-phase (Opp) constituted single-modal inputs. In-phase, water (In-W); in-phase, opposed-phase (In-Opp);﻿ in-phase, opposed-phase, water (In-Opp-W); and in-phase, opposed-phase, fat, water (In-Opp-F-W) constituted multi-modal inputs. For each experiment, the nnU-Net was trained and evaluated separately. Lastly, the segmentation results were analyzed quantitatively and statistically compared.

The workflow of our study is illustrated in Fig. 1.

### Study population

In this single-institution study, datasets of liver MRIs with a non-contrast 3 mm T1 vibe Dixon sequence were included from a pre-existing database of liver MRIs prospectively acquired in patients with suspected liver disease between 16/03/2016 and 08/02/2018, as previously published^19–21^. The following patients were excluded: those < 18 years of age, those denying consent, and those with prior focal liver lesions > 2 cm, prior liver resection or interventions, as well as patients with cholestatic liver disease. Clinical information was collected from the patients, including sex, age, body mass index (BMI), and etiology of chronic liver disease (CLD). The study was approved by the local ethics committee (Bern cantonal ethics committee, Bern, Switzerland) and was carried out in accordance with the principles of the Declaration of Helsinki. All patients gave written informed consent to participate in the study. The authors had full access to and take full responsibility for the integrity of the data.

### Magnetic resonance imaging

**Figure 2:**
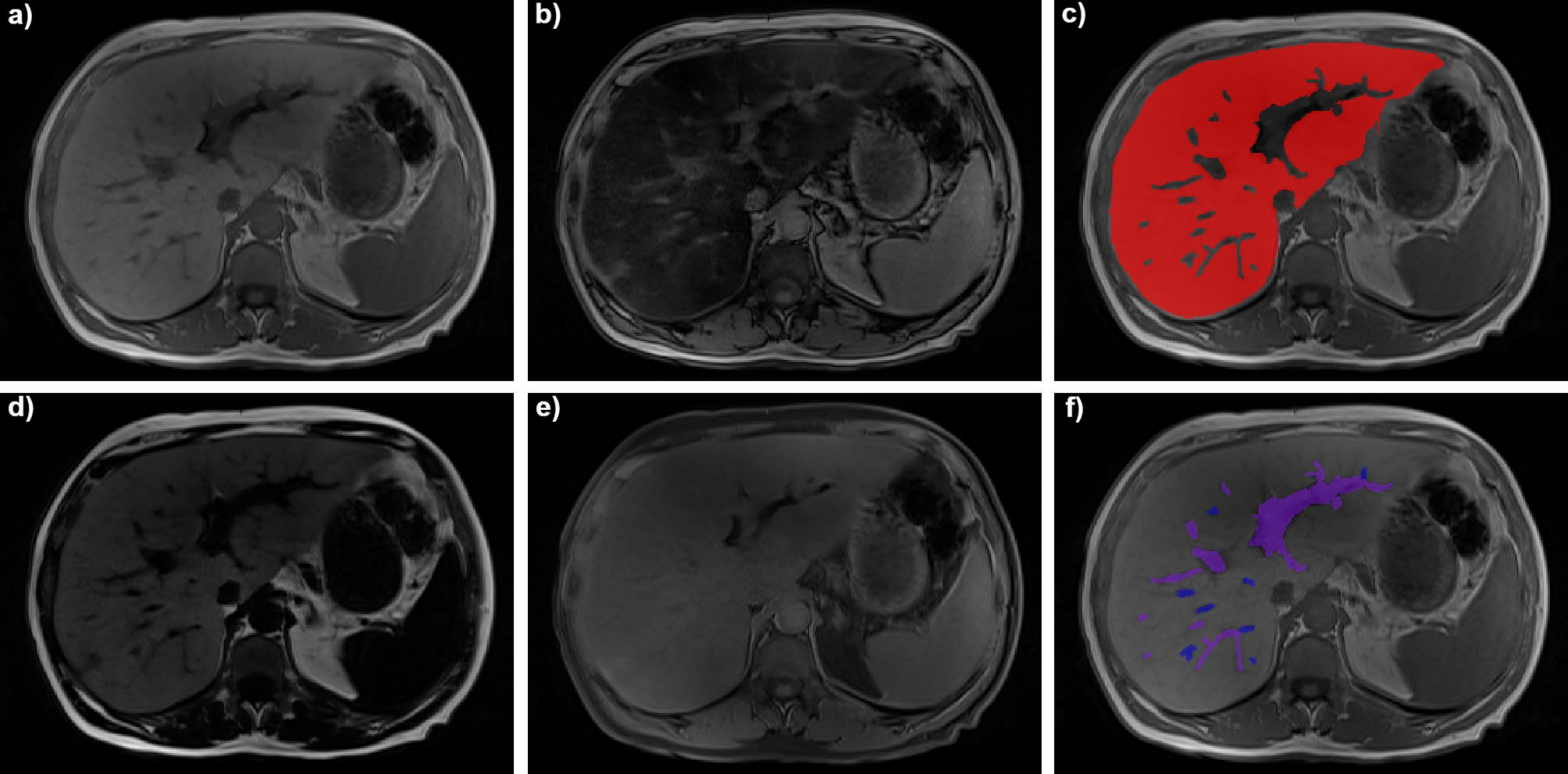
Example of a patient with a standard non-contrast T1 vibe Dixon acquisition of the liver. Please note the signal drop between (**a**) in-phase and (**b**) opposed-phase in this patient with severe steatosis, which can also be seen in (**d**) the fat and (**e**) water reconstructions. On the right-hand side, manual segmentation of (**c**) liver parenchyma (red) and (**f**) portal veins (purple) and hepatic veins (blue) on the in-phase.

All MRI datasets were acquired on a Siemens MAGNETOM Prisma^fit^ 3T scanner (Siemens Healthineers, Erlangen, Germany), using a 6-channel body coil. A standard non-contrast T1 vibe Dixon sequence was acquired, covering the whole liver by generating in-phase, opposed-phase, water, and fat reconstructions (Fig. 2). Images were acquired with a slice thickness of 3 mm, axial dimensions ranging from 210 × 320 to 270 × 320 pixels with pixel spacings ranging from 1.09375 × 1.09375 mm^2^ to 1.5625 × 1.5625 mm^2^ and 60 to 80 axial slices (Table 1). Cases with severe fat–water swaps (Dixon artifacts) were excluded. Cases with slight breathing artifacts were not excluded to obtain results that are comparable with real life clinical routine liver MRI acquisitions.

**Table 1:** Characteristics of MR imaging.

Characteristic	
Scanner model	Siemens Magnetom Prisma^fit^
Field strength (T)	3
Pixel spacing (mm^2^)	1.09375 × 1.09375 to 1.5625 × 1.5625
Slice thickness (mm)	3
Axial dimensions (pixel)	210 × 320—270 × 320
Axial slices, n	60–80

MR, magnetic resonance.

### Manual segmentation

The liver MRI datasets were manually labelled slice-by-slice for the outer liver border and the portal and hepatic veins by a trained reader with two years of experience in liver MRI (D.C.). The manual segmentation was performed using the medical software ITK-SNAP^22^ (version 3.8.0) on the in-phase images in the axial plane, as shown in Fig. 2. The results were reviewed by a board-certified abdominal radiologist with > 10 years’ experience with liver MRI (A.T.H.).

### Deep learning-based segmentation

**Figure 3:**
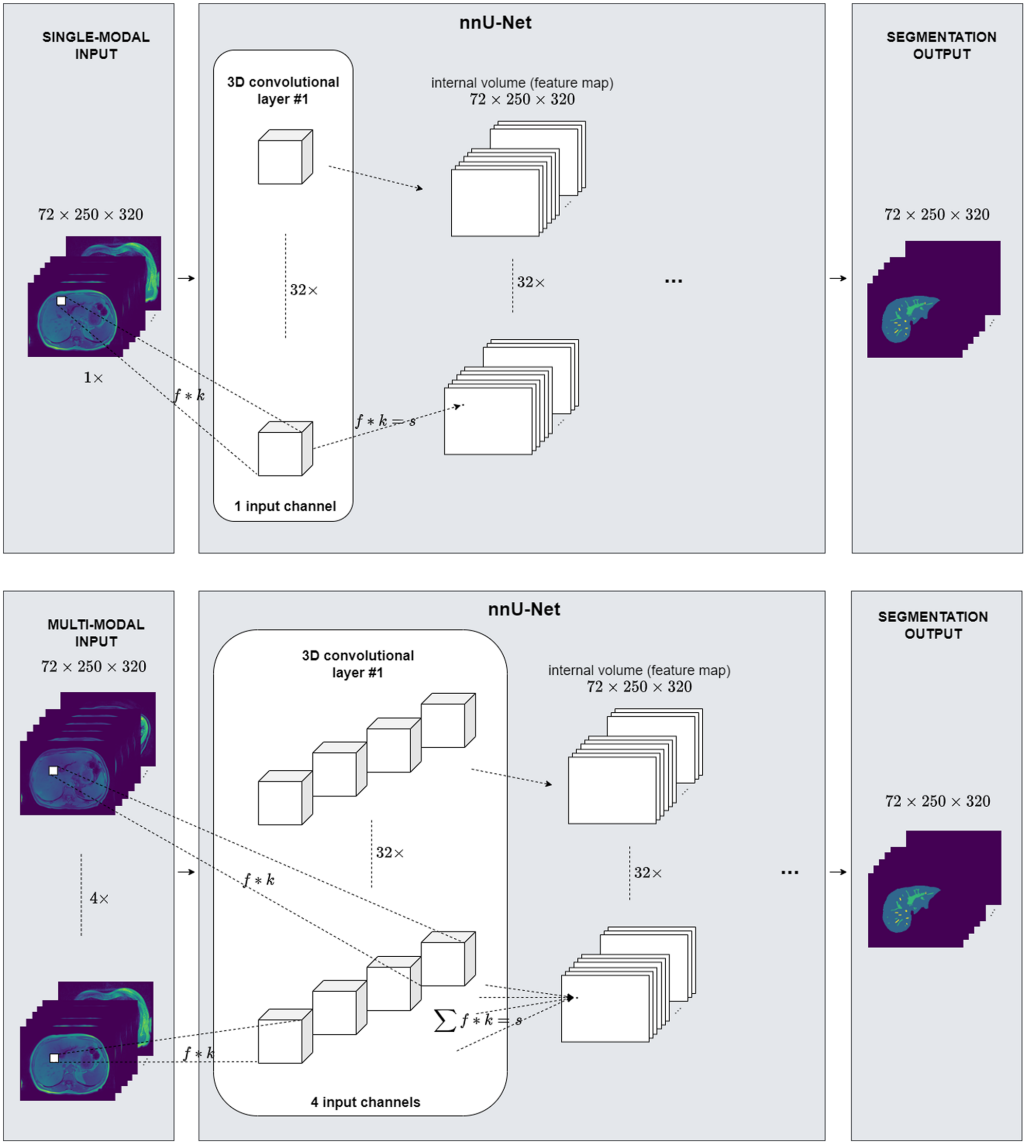
Processing of single-modal (top row) and multi-modal (bottom row) MRI sequence data at the input level of the 3D U-Net in use (nnU-Net^23^). The implementation uses 2D kernels at the first convolutional layer #1 since it treats the MRI sequences as anisotropic data.

The automated delineation of the liver and veins was implemented as a 3D voxel-wise multi-label classification task. Specifically, the nnU-Net^23^ framework was used as it offers a fully automated machine learning pipeline including data preprocessing, data augmentation, U-Net^12^-based neural network architecture optimization and data post-processing. To comply with the network input file format, all T1 vibe Dixon sequences in Digital Imaging and Communications in Medicine (DICOM) protocol were converted to the Neuroimaging Informatics Technology Initiative (NIfTI) format. The nnU-Net framework expects one or more 3D images as input and outputs a 3D segmentation mask of the same dimension (Fig. 3).

### Training and evaluation

The four T1 vibe Dixon in-phase, opposed-phase, water, and fat reconstruction imaging were used as isolated single-modal, as well as multi-modal inputs. A single-modal input referred to the case when only one reconstruction was used as an input to the neural network, whereas a multi-modal input referred to the case when two or more reconstructions were stacked together as an input to the neural network. We evaluated the impact of each type of input on the performance of the network model for the liver and vessel segmentation task. As for the neural network technique of processing multi-modal input, we used input-level fusion as in the work of Zhou et al.^24^. Figure 3 illustrates how the nnU-Net processed single-modal and multi-modal input at the first convolutional layer of the network.

The nnU-Net network was trained with the default setup as published by Isensee, Jager, et al.^23^. This included as loss function the combination of Dice loss and cross-entropy loss, the Adam optimizer with an initial learning rate of 3 × 10^–4^, and a learning rate scheduler that reduced the learning rate to at least 10^–6^ depending on the moving average of the training loss and the validation loss. For data augmentation, the following techniques were applied during training: random rotations, random scaling, random elastic deformations, gamma correction augmentation and mirroring. We did not make any changes to the architecture of the nnU-Net.

Nested cross-validation (NCV) was used to obtain a robust performance estimate on unseen test data. For each experiment, nnU-Net was trained 10 times on the liver MRI dataset with a leave-out test set of three different patients in every iteration (NCV outer loop). Performances on the test liver MRI datasets were averaged. For each training, the network was optimized on a NVIDIA GeForce RTX 3090 GPU for 150 epochs with a batch size of two using fivefold cross validation (NCV inner loop) following the approach by Isensee, Jaeger, et al.^23^.

### Quantitative analysis

The performance of the neural network to segment liver parenchyma, portal, and hepatic veins was compared between single- and multi-modal inputs. Dice similarity coefficient (DSC) was used to quantify the segmentation performance of the model. In addition, the average precision (AP) metric was calculated, as it ignores true negatives (negative background outside of the liver in the MRI field-of-view) and focuses on precision and recall. Kruskal–Wallis test with Dunn’s multiple comparison post hoc test was used to compare DSC between single- and multi-modal inputs. Significance level was chosen to be α = 0.05. All statistical analysis was performed with the Python ecosystem (Python 3.6.12, SciPy 1.5.4, scikit-learn 0.24.2, SimpleITK 2.0.2, Matplotlib 3.3.3). For the analysis of the segmentation performance in the central region versus the peripheral region of the liver, two points at the portal vein bifurcation and the hepatic vein confluence were manually set by a board-certified abdominal radiologist (A.T.H.). A sphere was defined with the center point being the midpoint of the two points and a radius of 75% of their distance. The region inside the sphere was considered as central region, the region outside the sphere was considered as peripheral region. The accuracy of the segmentation of portal veins, hepatic veins, and liver parenchyma was compared between the central and peripheral region.

## Results

### Patients

Liver MRI datasets from twenty female and ten male patients without fat–water swaps (Dixon artifacts) were included. Fifteen datasets were used from patients with chronic liver disease (50%) and fifteen from patients with no chronic liver disease (Table 2). Median patient age was 58.5 years (range 34–80 years) with a median weight of 80 kg (range 40–114) and a median BMI of 26.6 kg/m^2^ (range 15–45). Etiology of chronic liver disease was non-alcoholic fatty liver disease in eight patients, alcohol-related liver disease in three patients, and viral hepatitis in four patients. From the 15 patients with chronic liver disease, 9 patients had a liver cirrhosis. Median liver proton density fat fraction was 8%, ranging from 1.5–33.7%. Three datasets (10%) presented ghosting artifacts, in line with a realistic setting of routine abdominal MRI scans in a radiology department.

**Table 2:** Characteristics of patients.

Characteristic	
Sex	20 female / 10 male
Median age (range)	58.5 (34–80)
Median weight kg (range)	80 (40–114)
Median BMI kg/m^2^ (range)	26.6 (15.2–45.2)
Median PDFF % (range)	8.0 (1.5–33.7)
Chronic liver disease, n (CLD)	15
Etiology of CLD, n	8 NAFLD/NASH
	3 ARLD
	4 viral hepatitis
Liver cirrhosis, n	9

BMI, body mass index; PDFF, proton density fat fraction; CLD, chronic liver disease; NAFLD, non-alcoholic fatty liver disease; NASH, non-alcoholic steatohepatitis; ARLD, alcohol-related liver disease.

### Training runtime

The training of the network took roughly 4 h per dataset and fold, repeated in 10 iterations in 7 different single- and multi-modal experiments. Each iteration included 5 training folds following the fivefold cross validation training approach by nnU-Net. This resulted in a total of 70 different training datasets with a total training duration of 1400 h. Once a model was trained, liver parenchyma and vessel segmentation inference in one test liver MRI dataset was performed in 50 s using a NVIDIA GeForce RTX 3090 GPU, an AMD EPYC 7302 16-Core Processor CPU, and an IBM Spectrum Scale-based file system.

### Quantitative evaluation

**Figure 4:**
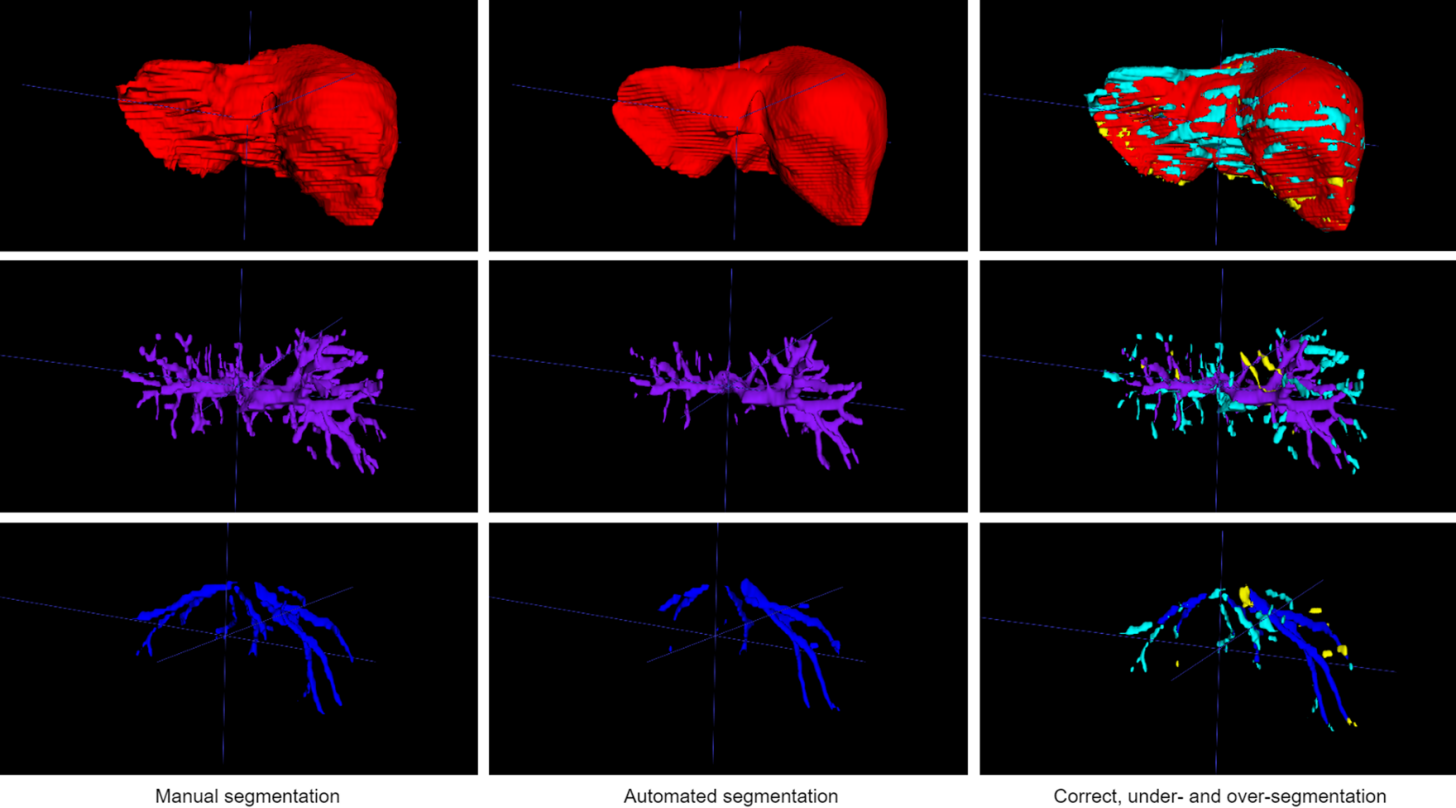
Example of an expert (first column) and automated (second column) segmentation of liver parenchyma (red), portal veins (purple), and hepatic veins (blue). The third column shows a visualization of the correct segmentation, as well as under-segmentation (cyan), and over-segmentation (yellow), as performed by the model with an in-phase sequence.

**Figure 5:**
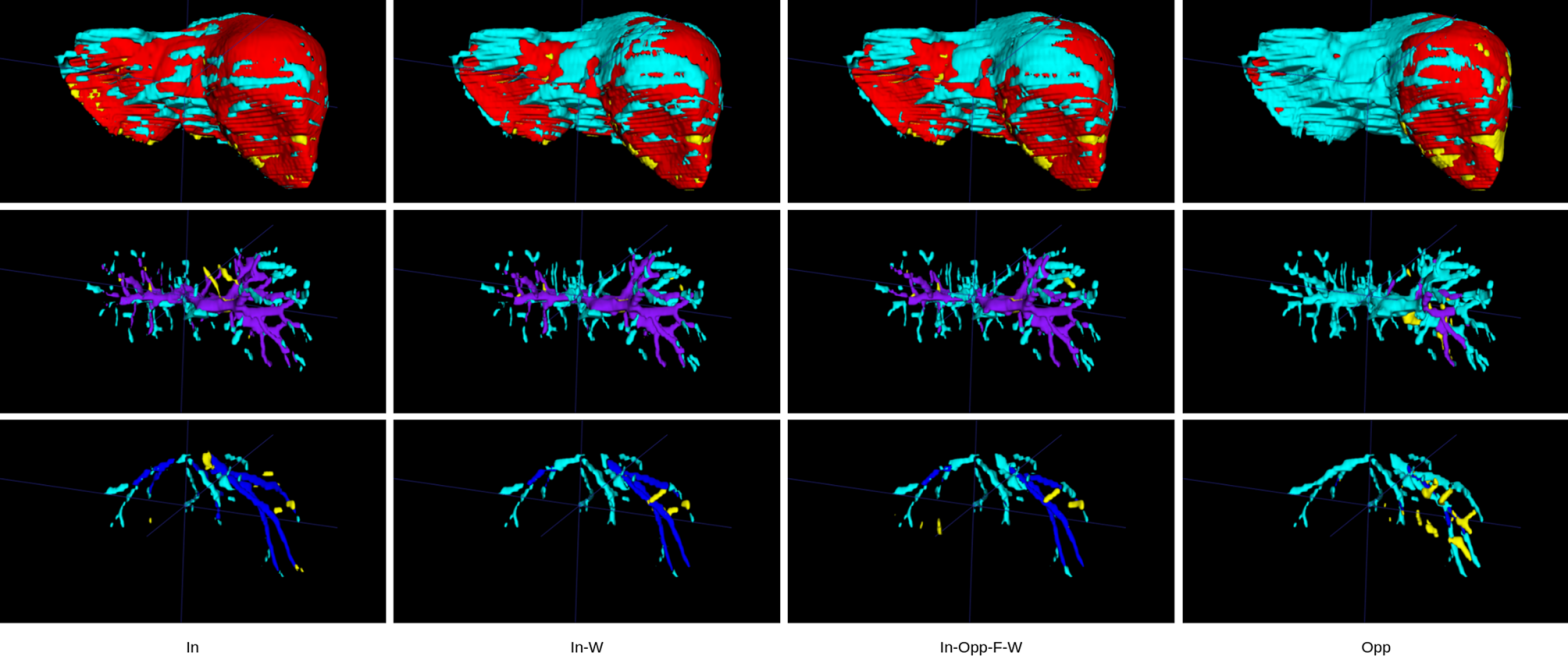
Comparison of liver segmentation performance with different model inputs: (1) single-modal in-phase (In) in the first column, (2) multimodal in-phase and water reconstruction in the second column (In-W), (3) multi-modal in-phase, opposed-phase, fat, and water reconstructions in the third column (In-Opp-F-W) and (4) single-modal opposed-phase (Opp) in the last column. The correct segmentation is shown in red for the liver parenchyma, purple for the portal veins, and blue for the hepatic veins. Under-segmentation is shown in cyan and over-segmentation in yellow, as performed by the model with an in-phase sequence.

**Figure 6:**
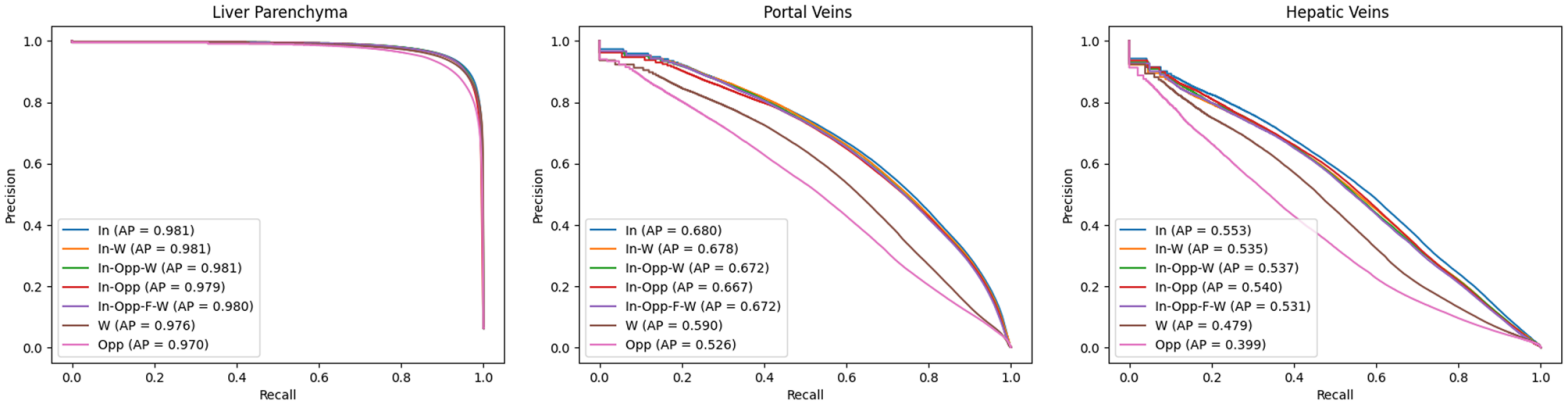
Precision-recall curves and average precision (AP) of automated liver parenchyma, portal veins, and hepatic veins segmentation on single- and multi-modal inputs. In-phase (In), water (W), and opposed-phase (Opp) constituted single-modal inputs. In-phase, water (In-W); in-phase, opposed-phase, water (In-Opp-W); in-phase, opposed-phase (In-Opp); and in-phase, opposed-phase, fat, water (In-Opp-F-W) constituted multi-modal inputs. Best viewed in screen.

Quantitative segmentation results from manual segmentation and neural network segmentation are illustrated in a representative case in Fig. 4, showing an excellent performance of liver parenchymal segmentation and a good delineation and differentiation between the portal and hepatic veins. The neural network trained with the single-modal in-phase reconstruction achieved the highest overall performance with an average DSC of 0.936 ± 0.02 for liver parenchyma, 0.634 ± 0.09 for portal veins, and 0.532 ± 0.12 for hepatic veins, as shown in Table 3. All single- and multi-modal inputs yielded comparable liver parenchyma segmentations without statistically significant differences (p = 0.09). In contrast, performance of the portal and hepatic veins segmentation was significantly lower when using the opposed-phase, as compared to the in-phase single-modal input (p < 0.001), while there was no significant difference between the in-phase and water-reconstruction input for portal (p = 0.331) and hepatic veins (p = 1.0) in the post hoc comparison (Table 4). There was no significant difference for liver, portal, and hepatic vessel segmentation between the in-phase single-modal input and the multi-modal input combining in-phase with opposed phase, fat, and water reconstructions (Fig. 5). Similar results were obtained by using the average precision metric, ignoring the true negative background outside of the liver in the MRI field of view, as demonstrated by the precision-recall curves in Fig. 6.

**Table 3:** Automated liver parenchyma, portal veins, and hepatic veins segmentation on single-modal and multi-modal T1 vibe Dixon acquisitions.

		In	In-W	In-Opp-W	In-Opp	In-Opp-F-W	W	Opp	p-value
Liver parenchyma	DSC	**0.936 ± 0.02**	0.935 ± 0.02	0.935 ± 0.02	0.934 ± 0.02	0.934 ± 0.02	0.931 ± 0.02	0.921 ± 0.03	0.090
	AP	**0.981 ± 0.01**	0.981 ± 0.01	0.981 ± 0.01	0.979 ± 0.01	0.980 ± 0.01	0.976 ± 0.02	0.970 ± 0.02	
Portal veins	DSC	**0.634 ± 0.09**	0.634 ± 0.08	0.631 ± 0.08	0.626 ± 0.08	0.632 ± 0.08	0.571 ± 0.14	0.505 ± 0.12	< 0.001
	AP	**0.680 ± 0.12**	0.678 ± 0.12	0.672 ± 0.12	0.667 ± 0.12	0.672 ± 0.12	0.590 ± 0.15	0.526 ± 0.13	
Hepatic veins	DSC	**0.532 ± 0.12**	0.523 ± 0.11	0.523 ± 0.11	0.525 ± 0.11	0.519 ± 0.11	0.475 ± 0.16	0.395 ± 0.12	< 0.001
	AP	**0.553 ± 0.12**	0.535 ± 0.12	0.537 ± 0.11	0.540 ± 0.12	0.531 ± 0.12	0.479 ± 0.16	0.399 ± 0.13	

Results are measured by Dice similarity coefficient (DSC) and average precision (AP) and presented as mean ± SD.

P-values were calculated using the Kruskal–Wallis test with Dunn’s multiple comparison post-hoc test. The single-modal neural network inputs are In, in-phase; W, water; Opp, opposed phase. The multi-modal neural network inputs are In-W, in-phase, water; In-Opp-W, in-phase, opposed-phase, water; In-Opp, in-phase, opposed-phase; In-Opp-F-W, in-phase, opposed-phase, fat, water. Best results are shown in bold.

**Table 4:** Post-hoc analysis of the performance of single-modal and multi-modal T1 vibe Dixon network inputs for automated liver parenchyma, portal veins, and hepatic veins segmentation.

			Liver parenchyma	Portal veins	Hepatic veins
In-phase	vs	Opposed-phase	0.129	** < 0.001**	** < 0.001**
In-phase	vs	Water	1	0.331	1
In-phase	vs	In-phase, water	1	1	1
In-phase	vs	In-phase, opposed-phase	1	1	1
In-phase	vs	In-phase, opposed-phase, water	1	1	1
In-phase	vs	In-phase, opposed-phase, water, fat	1	1	1
Opposed-phase	vs	Water	1	0.365	0.112
Opposed-phase	vs	In-phase, water	0.310	** < 0.001**	**0.002**
Opposed-phase	vs	In-phase, opposed-phase	1	**0.001**	**0.002**
Opposed-phase	vs	In-phase, opposed-phase, water	0.256	** < 0.001**	**0.003**
Opposed-phase	vs	In-phase, opposed-phase, water, fat	0.302	** < 0.001**	**0.004**
Water	vs	In-phase, water	1	0.403	1
Water	vs	In-phase, opposed-phase	1	0.921	1
Water	vs	In-phase, opposed-phase, water	1	0.641	1
Water	vs	In-phase, opposed-phase, water, fat	1	0.629	1
In-phase, water	vs	In-phase, opposed-phase	1	1	1
In-phase, water	vs	In-phase, opposed-phase, water	1	1	1
In-phase, water	vs	In-phase, opposed-phase, water, fat	1	1	1
In-phase, opposed-phase	vs	In-phase, opposed-phase, water	0.444	1	1
In-phase, opposed-phase	vs	In-phase, opposed-phase, water, fat	1	1	1
In-phase, opposed-phase, water	vs	In-phase, opposed-phase, water, fat	1	1	1

The p-values were calculated with the Kruskal–Wallis test with Dunn’s multiple comparison post-hoc test using the Dice similarity coefficients reported in Table 3. Any values below the significance level α = 0.05 are highlighted in bold. The single-modal neural network inputs are in-phase; water; and opposed phase. The multi-modal neural network inputs are in-phase, water; in-phase, opposed-phase; in-phase, opposed-phase, water; and in-phase, opposed-phase, fat, water.

**Figure 7:**
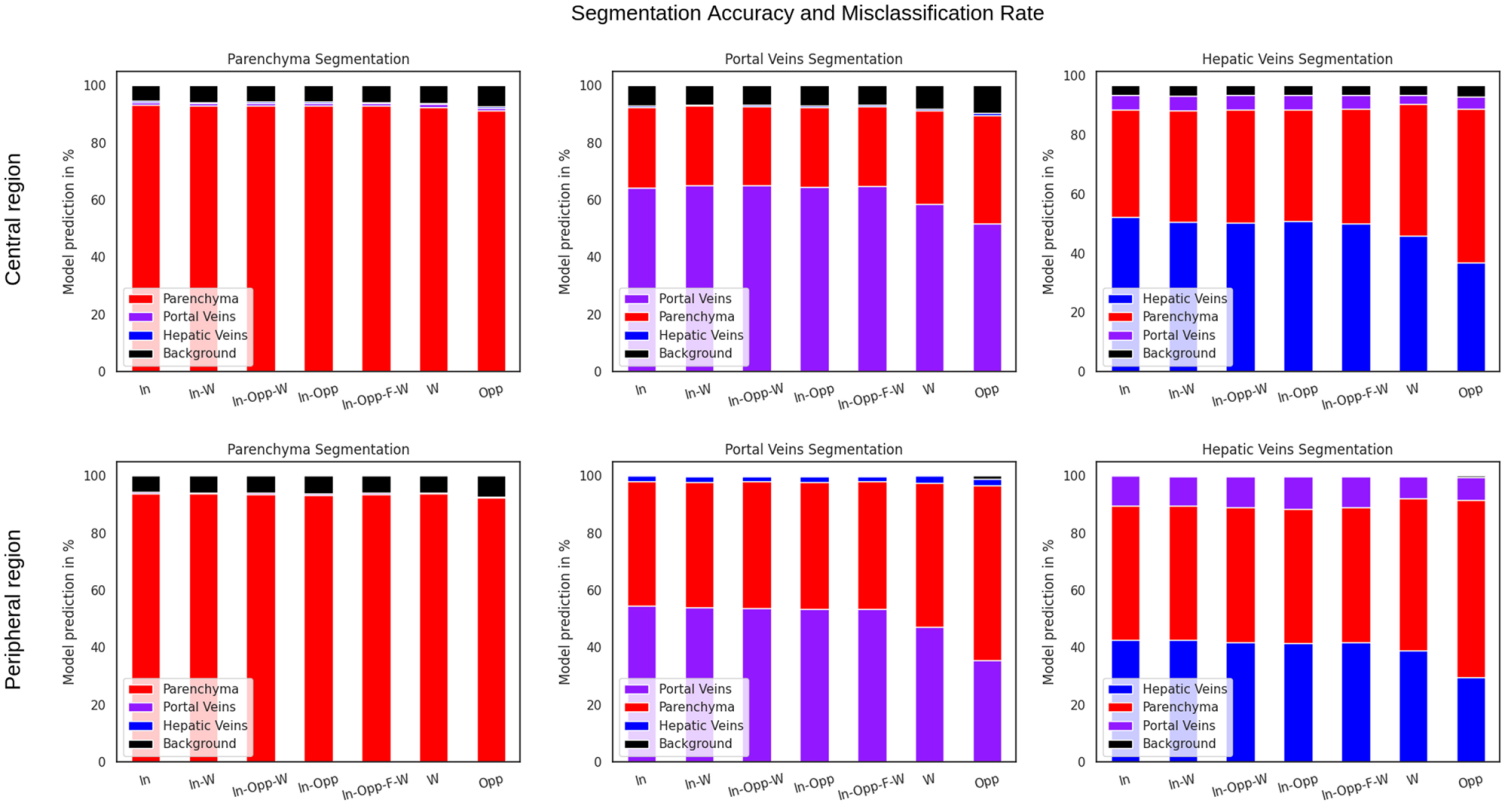
Model segmentation accuracy and misclassification rate for liver parenchyma segmentation (left), portal veins segmentation (middle), and hepatic veins segmentation (right). The results are shown for the central region of the liver (upper row) and the peripheral region of the liver (bottom row). In-phase (In), water (W), and opposed-phase (Opp) constituted single-modal inputs. In-phase, water (In-W); in-phase, opposed-phase, water (In-Opp-W); in-phase, opposed-phase (In-Opp); and in-phase, opposed-phase, fat, water (In-Opp-F-W) constituted multi-modal inputs. Liver parenchyma segmentation is shown in red, portal veins segmentation in purple, hepatic veins segmentation in blue, and background segmentation in black. The colors are identical to Figs. 4 and 5. Best viewed in screen.

The accuracy of parenchymal segmentation with the single-modal in-phase reconstruction was 93% in the central region and 94% in the peripheral region. The accuracy of portal veins segmentation was 64% in the central region and 55% in the peripheral region. The accuracy of hepatic veins segmentation was 52% in the central region and 43% in the peripheral region, as shown in Fig. 7. If only the vascular voxels were analyzed, without the liver parenchyma, accuracy for differentiation between portal and hepatic veins was 99% for portal veins and 97% for hepatic veins in the central region and slightly lower in the peripheral region (91% for portal veins and 80% for the hepatic veins).

## Discussion

To the best of our knowledge, this is the first study that uses a 3D neural network (nnU-Net^23^) for automated liver parenchyma, portal veins, and hepatic veins segmentation on non-contrast T1 vibe Dixon liver MRIs. The performance of liver parenchyma segmentation was excellent, when compared with a manual slice-by-slice segmentation as the gold standard. The delineation of hepatic and portal veins was highly accurate, and voxels were rarely classified to the incorrect venous system. Based on this convolutional neural network, segmentation of liver parenchyma, portal, and hepatic veins was possible on a standard non-contrast T1 vibe Dixon sequence in less than one minute.

Our liver parenchyma results are comparable with existing literature, as shown in Table 5. Kavur et al.^14^ presented an nnU-Net-based evaluation for liver parenchyma segmentation with a DSC of 0.95, which is very similar to our DSC of 0.94 for the single-modal in-phase input. However, Kavur et al. did not separate the liver veins from the parenchyma, so their algorithm only segmented the outer liver by ignoring the hepatic and portal veins. Kart et al.^13^ achieved even slightly higher DSCs of 0.97–0.98 on non-contrast T1 weighted images but analyzed in healthy volunteers and not in patients with chronic liver disease and by ignoring the liver veins as well. Ivashchenko et al.^25^ proposed a liver segmentation workflow based on contrast-enhanced multi-phase MRIs, resulting in a DSC of 0.95 for liver parenchyma. Ivashchenko et al.^26^ also studied the feasibility of liver vessel segmentation on contrast enhanced MRIs using a DL-based method, resulting in a median DSC of 0.60 for portal veins and 0.65 for hepatic veins. While our result for portal veins is slightly better (0.66), the results for the hepatic veins was slightly lower (0.55), which may be explained by the fact that we used non-contrast T1 vibe Dixon sequences and our manual segmentation labelled even the small peripheral hepatic veins, which were difficult to detect for the neural network. Other groups tried to segment liver veins on T1 weighted images with a non-DL-based approach using thresholding and filtering, but without separating between portal and liver veins^18^. Other non-DL-based approaches were published for liver vessel segmentation on CT and MRI images^15^, contrast-enhanced T1 weighted images^16^, and Fast Imaging with Steady-state Precession (FISP) sequences^17^, some without differentiation between portal and liver veins.

**Table 5:** Quantitative performance comparison between MRI-based liver parenchyma and liver veins segmentation methods.

	Model	Liver parenchyma	Portal veins	Hepatic veins		Dataset			
		Mean ± SD	Median (IQR)	Median (IQR)	Contrast enhanced	MRI characteristrics	Subjects	Name	Test size, n
Kavur et al.^14^	nnU-Net	0.954 ± 0.01	-	-	No	T1-DUAL in-phase, opposed-phase	healthy	CHAOS	20
Kart et al.^13^	nnU-Net	0.972 ± 0.02	-	-	No	T1 Dixon water	healthy	UKBB	200
Kart et al.^13^	nnU-Net	0.984 ± 0.01	-	-	No	T1 Dixon in-phase, opposed-phase, water, fat	healthy	GNC	200
Ivashchenko et al.^25^	workflow	0.950 ± 0.01	-	-	Yes	multiphase T1 mDixon water	lesions	private	15
Ivashchenko et al.^26^	DVNet	-	0.603 (0.08)	0.647 (0.05)	Yes	T1 mDixon	tumors	private	20
Ours	nnU-Net	0.936 ± 0.02	0.659 (0.11)	0.548 (0.16)	No	T1 Dixon in-phase, opposed-phase, water, fat	lesions	private	30

Summary of papers on MRI liver and vessels segmentation. Our study is the first to evaluate a 3D convolutional neural network (nnU-Net^23^) for automated segmentation on non-contrast T1 vibe Dixon liver MRIs with lesions.

All scores are in Dice metric. Liver parenchyma results are shown as mean ± SD. The portal veins and hepatic veins segmentation results are compared using median and interquartile range (IQR). The MRI dataset used by each method is summarized.

MRI, magnetic resonance imaging; CHAOS, combined healthy abdominal organ segmentation challenge; UKBB, UK Biobank; GNC, German National Cohort.

A multi-modal input from combined T1, T2, and FA sequences^27^ or the combination of FLAIR, T1, T1c, and T2^28^ increased the performance of neural network models for neuro-imaging segmentations. However, the use of different MRI sequences needs spatial co-registration^24, 27^, which is easier to perform on neuro-imaging studies, whereas liver MRIs commonly contain more motion artefacts. The T1 vibe Dixon sequence used for this study has an optimal fat–water separation^29^ without the need for spatial co-registration and provides a high level of contrast between veins and liver parenchyma. However, the combination of in-phase, opposed-phase, fat, and water inputs did not increase the performance of the liver and veins segmentation. While the best performance was achieved with a single-modal in-phase input, the lowest performance was achieved with a single-modal opposed-phase input. A possible explanation for this observation is the occurrence of chemical shift artifacts (India ink artifacts) on opposed-phase images, occurring at fat–water boundaries, such as the outer liver border or the vessel-parenchyma interfaces. Combination of in- and opposed-phase images as a multi-modal input therefore showed a lower performance than the isolated single-modal in-phase input. As fat and water reconstructions are calculated based on the in- an opposed-phase images, it is no surprise that the addition of those reconstructions did not increase the model performance. In patients with significant liver steatosis, the contrast between the liver parenchyma and liver veins will be inverted, as compared to the in-phase acquisition. The median proton density fat fraction of the MRI liver datasets in this study was 8.0% and ranged from 1.5% to 33.7%. A training on a dataset including more patients with liver steatosis should be performed to test whether the convolutional neural network would profit of a multi-modal in- and opposed-phase input in those patients.

This study has several limitations. The used dataset was relatively small, and a larger dataset may result in better portal and hepatic veins segmentation. However, the performance of the hepatic parenchyma segmentation was comparable to the performance of DL-algorithms trained on larger datasets^13^. Another inherent limitation is the slice thickness of 3 mm of the used standard T1 vibe Dixon MRI sequences. The performance of the DL-algorithm may improve when using 3D T1 sequences with a smaller slice thickness. However, the T1 vibe Dixon sequences are very robust, fast, and widely available. Finally, another limitation is that MRI acquisitions were all performed on 3T MRI scanners from a single manufacturer. This resulted in a relatively homogeneous data set and the study should be extended and validated on different scanners with different field strengths.

In conclusion, neural network segmentation of liver veins and parenchyma on non-contrast T1 vibe Dixon is highly effective. The best performance was achieved with a single-modal in-phase input for automated liver parenchyma segmentation and good differentiation between portal and hepatic veins.

## Data Availability

Data generated or analyzed during the study are available from the corresponding author by request.
